# Effects of Intraoperative Neural Tunnel in Protecting Recurrent Laryngeal Nerve: Experiences in Open, Trans Breast, and Transoral Endoscopic Thyroidectomy

**DOI:** 10.3389/fonc.2022.779621

**Published:** 2022-02-23

**Authors:** Xing Yu, Yujun Li, Chang Liu, Yuancong Jiang, Zhaodi Liu, Qionghua He, Yong Wang, Ping Wang

**Affiliations:** ^1^ Department of Thyroid Surgery, The Second Affiliated Hospital, Zhejiang University School of Medicine, Hangzhou, China; ^2^ College of Medicine, Zhejiang University, Hangzhou, China

**Keywords:** recurrent laryngeal nerve, endoscopic thyroidectomy, energy-based devices, nerve thermal injury, inflammatory responses

## Abstract

**Background:**

Energy-based devices (EBDs) increase the risks of thermal nerve injuries. This study aimed to introduce a surgical strategy of intraoperative neural tunnel protecting (INTP) for evaluating the effect in reducing the incidence of recurrent laryngeal nerve (RLN) damage in open, trans breast, and transoral endoscopic thyroidectomy.

**Methods:**

INTP strategy was introduced: a tunnel was established and protected by endoscopic gauze along the direction of the nerve. A total of 165, 94, and 200 patients with papillary thyroid carcinoma (PTC) were to use INTP in respectively open, trans breast, and transoral endoscopic thyroidectomy as the INTP group. Additionally, 150, 95, and 225 patients who received the same methods without INTP were enrolled in the control group. Ipsilateral thyroidectomy or total thyroidectomy, and central compartment dissection were performed on the enrolled patients.

**Results:**

Clinicopathologic characteristics, surgical outcomes, and surgical complications were similar between the INTP group and the control group in open, trans breast, and transoral endoscopic thyroidectomy. The incidences of electromyography (EMG) changes in the INTP group were lower as compared to the control group in trans breast endoscopic thyroidectomy (*p* < 0.05). The incidence of postoperative hoarse in the INTP group was lower as compared to the control group in open and transoral endoscopic thyroidectomy (*p* < 0.05). Postoperative calcium levels (*p* < 0.01) were significantly higher, and the white blood cells (*p* < 0.05) and C-reactive protein levels (*p* < 0.01) were significantly decreased in the INTP group compared with the control group in transoral endoscopic thyroidectomy.

**Conclusions:**

This was the first instance of the INTP strategy being introduced and was found to be an effective method for protecting the RLN in open, trans breast, and transoral endoscopic thyroidectomy. Additionally, INTP helped protect other important tissues such as the parathyroid glands in transoral endoscopic thyroidectomy as well as in reducing postoperative inflammatory responses.

## Introduction

Paralysis of the vocal cord (VC) is one of the most frequent and serious complications following thyroidectomy surgeries (1). Studies have reported the incidence of transient and permanent VC paralysis to be approximately 3% to 5% and 1% to 2%, respectively, post-thyroidectomy surgery (2, 3). Studies by Huang et al. (4) and Hayami et al. (5) demonstrated that the recurrent laryngeal nerve (RLN) is at higher risk of developing thermal damage caused by energy-based devices (EBDs). Thermal injury induced by EBDs is considered to be one of the common mechanisms of RLN injury (6). There has been an increase in the incidence of iatrogenic RLN thermal injury over the past 10–15 years (7).

Studies have been performed to protect the nerves from EBD-induced thermal damage, which had defined safety parameters, such as lateral thermal spread (8). However, lots of other risk factors are still unclear. A previous study by our group found that thermal liquid–gas flow was a risk factor for thermal injury to the RLN (9). In the current study, we introduced a new strategy of intraoperative neural tunnel protecting (INTP) for evaluating the effects of INTP in reducing the incidences of RLN damages in open, trans breast, and transoral endoscopic thyroidectomy.

## Materials and Methods

### Patient Enrollment

**Figure 1:**
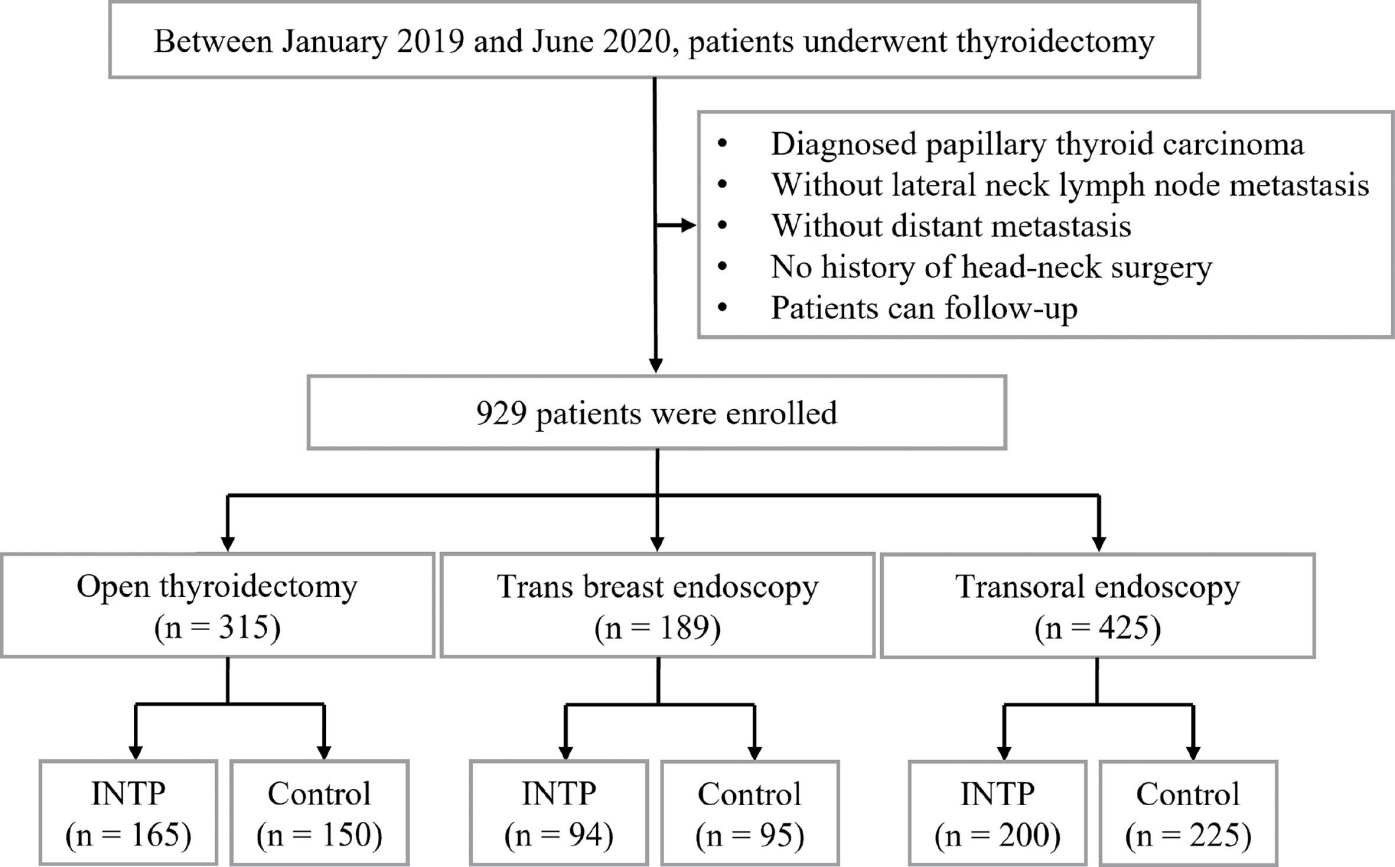
Study flowchart: enrollment and group assignment of the retrospective study.

This study was approved by the ethics committee of the Second Affiliated Hospital of Zhejiang University School of Medicine. Between January 2019 and June 2020, a total number of 929 consenting patients with papillary thyroid carcinoma (PTC) who underwent thyroidectomy and central compartment dissections (CCDs) were enrolled in this retrospective study at the Thyroid Surgery Department of the Second Affiliated Hospital, Zhejiang University School of Medicine. A total of 165, 94, and 200 enrolled patients were performed with the method of INTP in open, trans breast, and transoral endoscopic thyroidectomy, respectively, as the INTP group. Additionally, 150, 95, and 225 patients who received conventional methods without INTP in open, trans breast, and transoral endoscopic thyroidectomy, respectively, were enrolled as the control group (**Figure 1**). Patients’ clinical characteristics such as age, gender, body mass index (BMI), tumor size, multiple lesion ratio, and Hashimoto’s thyroiditis ratio were compared between the INTP group and the control group.

### Procedure of Thyroidectomy and Lymph Node Dissection

Trans breast and transoral endoscopic thyroidectomy were applied the same as open surgery. The trans breast approach has been used in multiple medical centers, whose operating steps had been described previously (10). Transoral endoscopic thyroidectomy has been briefly described below. Epinephrine solution was injected from the vestibule to the anterior of the neck at the subcutaneous layer for space creation. A 10-mm incision for the camera port was made in the middle of the vestibule and frenulum. Another two 5-mm trocars were applied through the mucosa incision at the level of the first premolars for auxiliary use. A 30° angled camera was then advanced through the 10-mm port. Flap dissection and thyroid vessel dividing were implemented using ultrasonic coagulation devices (harmonic scalpel (HS), Ethicon Endo-Surgery, Cincinnati, OH, USA). The inferior thyroid arteries and middle thyroid veins were isolated and coagulated by HS. Dissection of the central compartment lymph nodes was carried out. This also included lymph nodes in the prelaryngeal, pretracheal, and paratracheal areas. Following the exposure and dissection of the RLN, the lobe was completely resected using the ultrasound device and pliers in the control group. INTP was applied in the open, trans breast, and transoral endoscopic thyroidectomy in the experimental group.

### Introduction of Intraoperative Neural Tunnel Protecting

**Figure 2:**
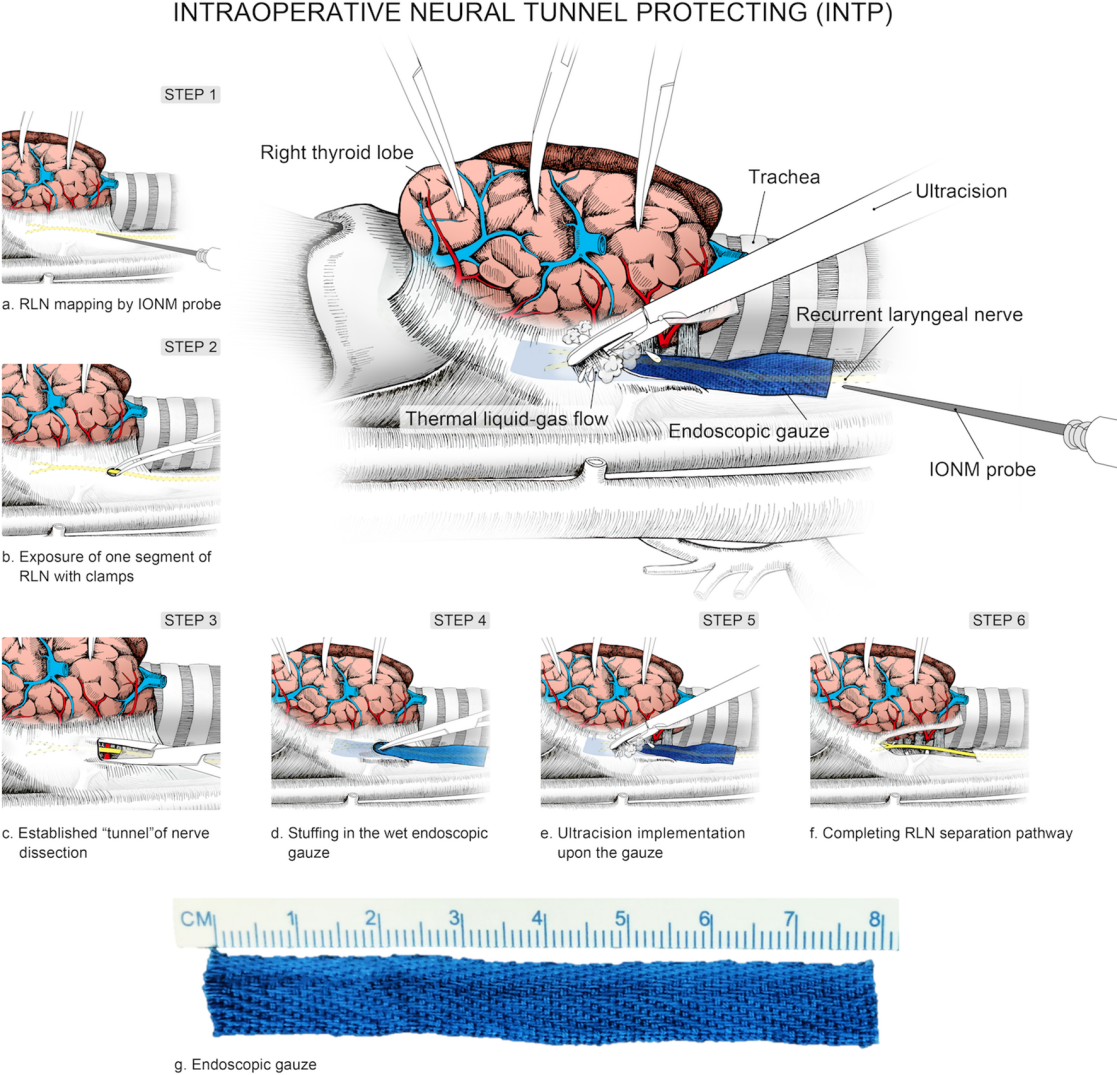
Schematic diagram of intraoperative nerve tunnel protecting (INTP).

**Figure 3:**
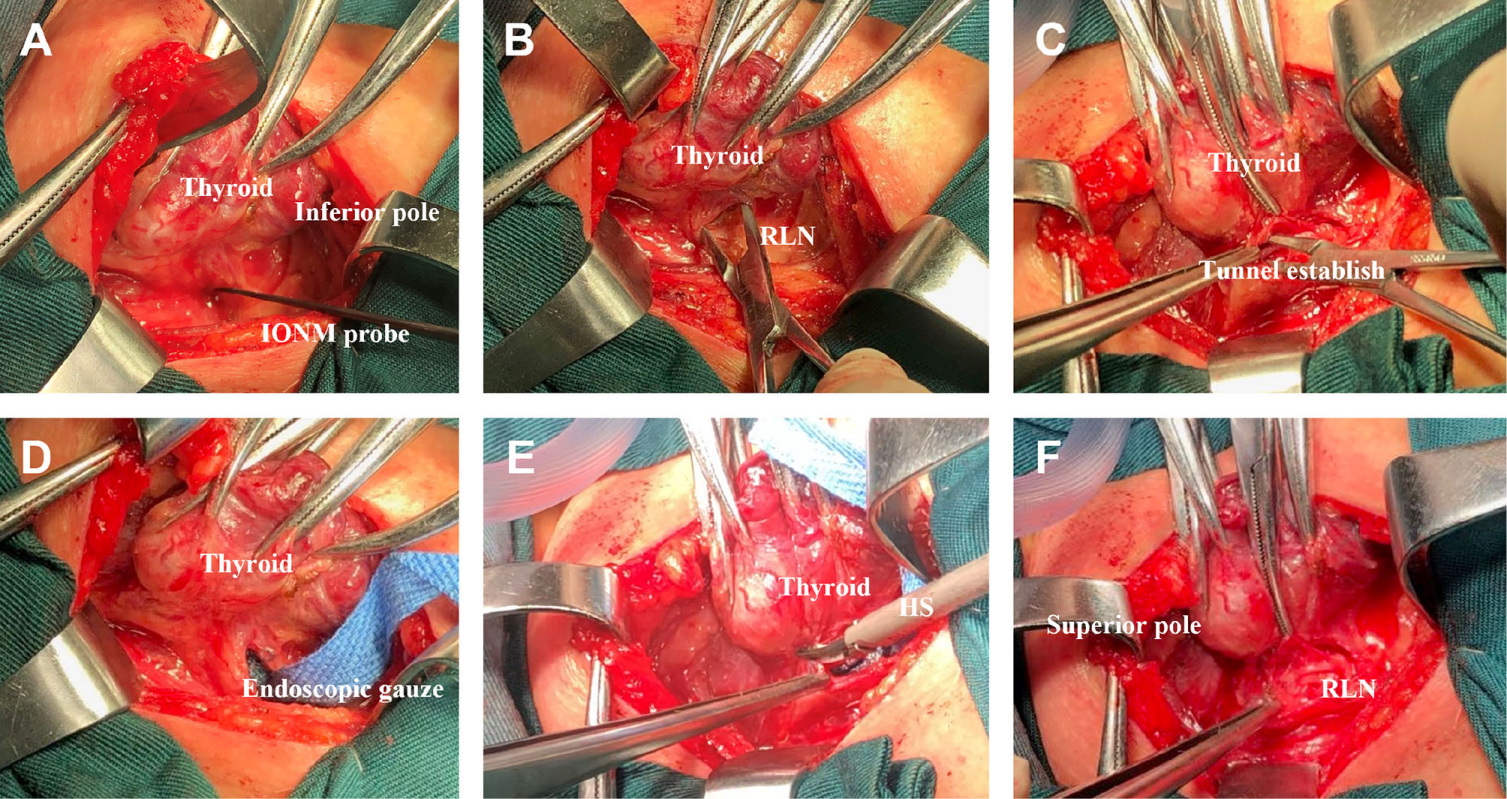
Intraoperative nerve tunnel protecting (INTP) strategy was applied in open thyroidectomy. **(A)** Inferior pole of thyroid lobe was exposed, and the recurrent laryngeal nerve (RLN) was mapped by intraoperative neural monitoring (IONM) probe. **(B)** Expose one segment of RLN with clamps. **(C)** A tunnel was established along the direction of RLN with clamps. **(D)** Endoscopic gauze was stuffed in the tunnel and covered the surface of RLN. **(E)** Gauze protects RLN from thermal liquid–gas when harmonic scalpel (HS) was used to dissect non-neural tissues. **(F)** The “tunnel” was extended by a constant deepening of the gauze and gradually completed the separate pathway.

**Figure 4:**
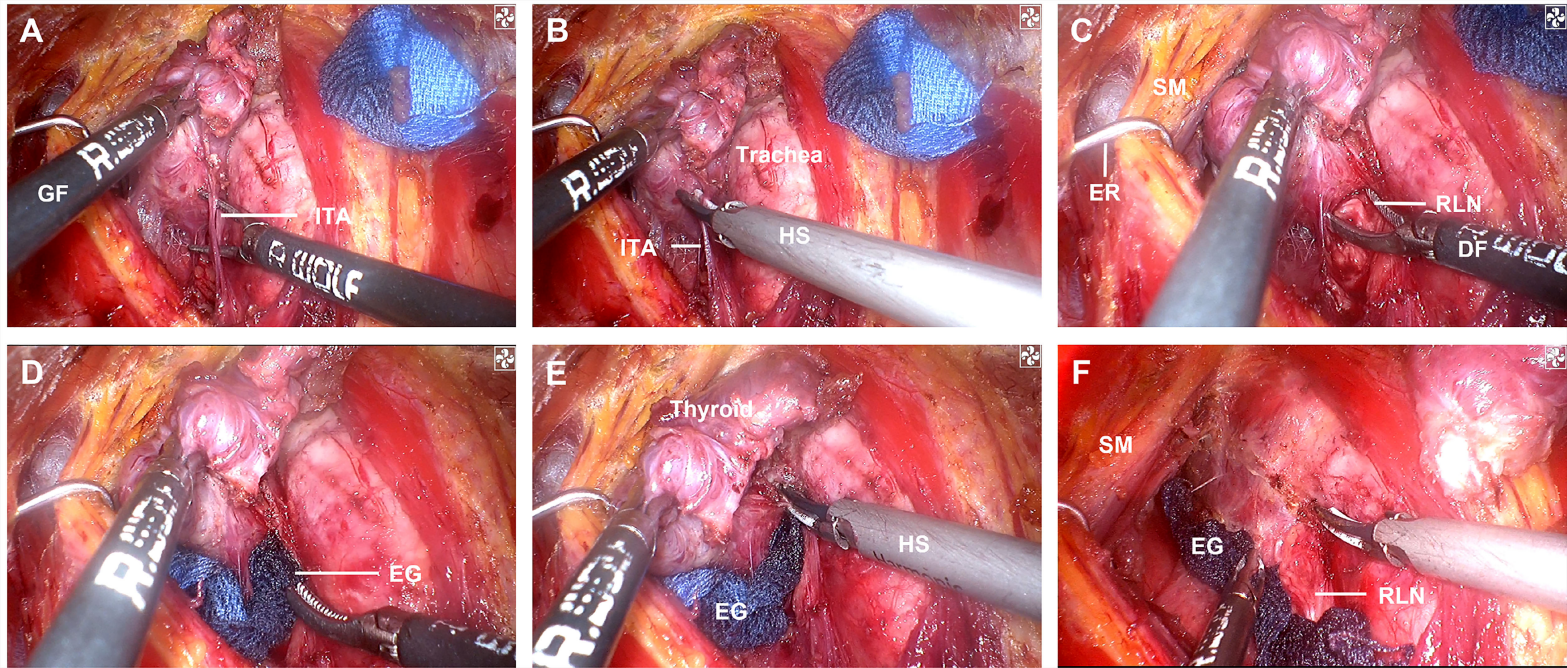
INTP strategy was applied in trans breast endoscopic thyroidectomy. **(A)** Expose the inferior pole of thyroid lobe. **(B)** Expose one segment of RLN. **(C)** Establish the “neural tunnel”. **(D)** Endoscopic gauze was stuffed. **(E)** UltraCision implementation upon the gauze. **(F)** Completing RLN separation pathway. GF, grasp forceps; ITA, inferior thyroid artery; HS, harmonic scalpel; SM, strap muscles; DF, dissection forceps; RLN, recurrent laryngeal nerve; EG, endoscopic gauze; INTP, intraoperative nerve tunnel protecting.

**Figure 5:**
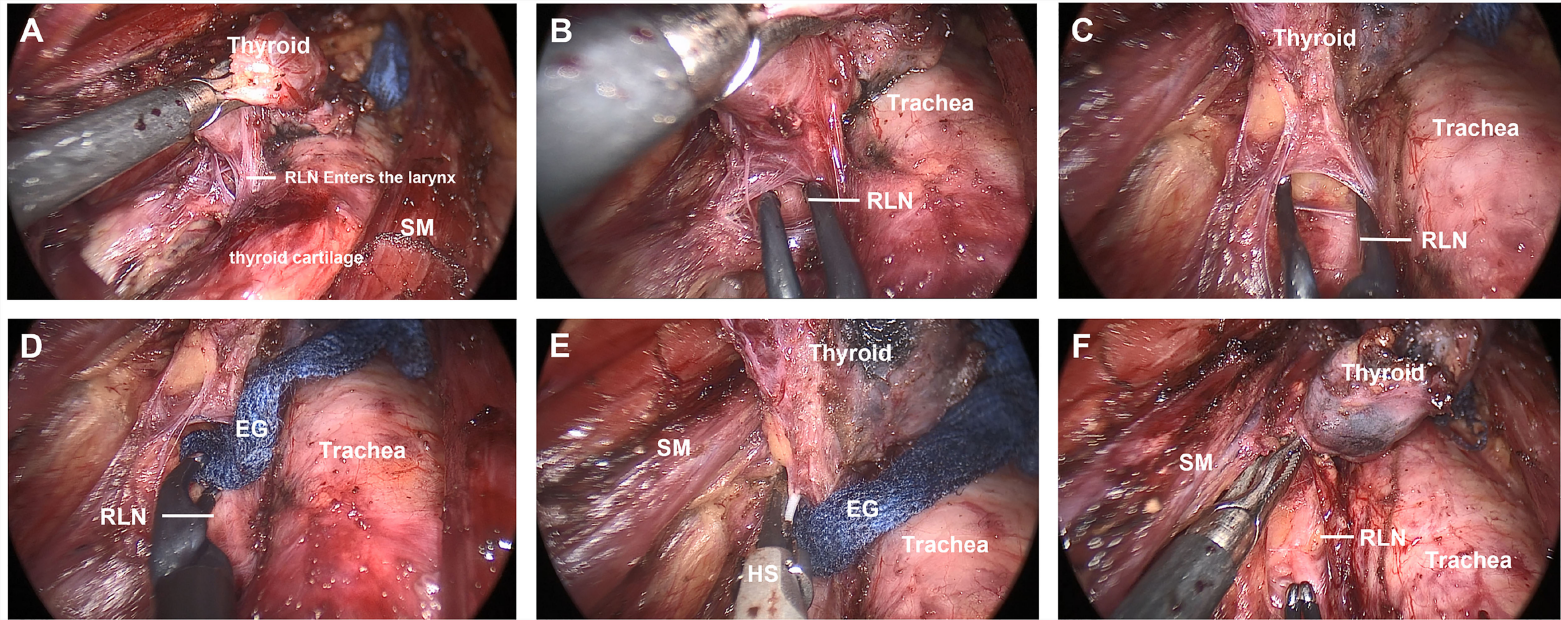
INTP strategy was applied in transoral endoscopic thyroidectomy. **(A)** Exposure of superior pole of thyroid lobe. **(B)** Exposure of one segment of RLN. **(C)** Establish the “neural tunnel.” **(D)** Endoscopic gauze was stuffed. **(E)** UltraCision implementation upon the gauze. **(F)** Completion of RLN separation pathway. INTP, intraoperative nerve tunnel protecting; RLN, recurrent laryngeal nerve.

A practical and programmed procedure of INTP was introduced for nerve protecting. *Step 1*: The inferior pole of the thyroid lobe was exposed, and the RLN was mapped by intraoperative neural monitoring (IONM) in the paratracheal region. *Step 2*: The RLN was exposed and identified with clamps. *Step 3*: A tunnel was established along the direction of the RLN with clamps. *Step 4*: Endoscopic gauze was stuffed in the tunnel and covered the surface of the RLN. *Step 5*: Gauze provided a separation barrier between thermal liquid–gas and the RLN when the HS was used to dissect non-neural tissues adjacent to the nerve. *Step 6*: The “tunnel” was extended by a constant deepening of the gauze and gradually completed the separate pathway (**Figure 2**). In brief, the meaning of INTP is a tunnel that was established along the direction of the nerve and then protected by endoscopic gauze. Instead of traditional white, endoscopic gauze was designed as blue for better differentiation from surrounding tissue so that blue becomes purple rather than fresh-colored pink when soaked with blood and fluid. The size was eventually identified as 80 mm × 15 mm × 1 mm after multiple attempts for the easiest use during endoscopic practice and the best protective effects of nerve protection. The procedures of INTP were introduced in open thyroidectomy (**Figure 3**), trans breast endoscopic thyroidectomy (**Figure 4**), and transoral endoscopic thyroidectomy (**Figure 5**). In the present study, intermittently IONM was performed with the standard four-step procedure. All electromyography (EMG) amplitudes (V1–R1–R2–V2) were obtained and recorded during vagus nerve stimulation before thyroid dissection (V1) and after thyroidectomy (V2), and RLN stimulation at initial identification (R1) and after dissection (R2) (11).

### Postoperative Controls

Preoperative laryngoscope, parathyroid hormone (PTH), and calcium were recorded 3 days before surgery. Postoperative laryngoscope, laboratory tests including PTH, calcium, white blood cell (WBC), and C-reactive protein (CRP) were examined on the day after the operation. Patients who had symptoms of numbness or convulsions in limbs received 2 g of intravenous calcium gluconate. An oral calcium supplement was administered to relieve symptoms of hypocalcemia.

There was a postoperative review of patients at our medical center every month for 3 months post-surgery. The levels of thyroid function, serum calcium, and PTH were tested; and the thyroid-stimulating hormone (TSH) inhibition therapy strategy follows the 2015 American Thyroid Association (ATA) thyroid nodule guideline. When the TSH level was stably adjusted to the target range, we conducted patient follow-up every 6–12 months, thyroid function and cervical B ultrasound were included each time.

### Statistical Analysis

The results are presented as number (%) and average ± SD, when appropriate. Data were analyzed by one-way ANOVA, Welch’s ANOVA, Student’s t-test, the χ^2^ test, and Fisher’s exact test using SPSS 20.0 software (SPSS Inc., Chicago, IL, USA). A *p*-value of less than 0.05 was considered to be statistically significant.

## Results

### Clinicopathologic Characteristics

The clinicopathologic characteristics are summarized in **Tables 1**–**3**. The INTP groups consisted of 165 (open thyroidectomy), 94 (trans breast endoscopic thyroidectomy), and 200 (transoral endoscopic thyroidectomy) patients. The control groups enrolled 150 (open thyroidectomy), 95 (trans breast endoscopic thyroidectomy), and 225 (transoral endoscopic thyroidectomy) patients. The patients’ age, sex ratio, and BMI were similar in the INTP and control groups in open, trans breast, and transoral endoscopic thyroidectomy. There was no difference between the 2 groups in the max tumor size, multiple lesion ratio, and Hashimoto’s thyroiditis ratio in open, trans breast, and transoral endoscopic thyroidectomy. The total thyroidectomy ratio and bilateral CCD ratio were similar between the two groups in open, trans breast, and transoral endoscopic thyroidectomy.

**Table 1:** Clinicopathologic characteristics in open thyroidectomy.

	INTP group (n = 165)	Control group (n = 150)	*p*-Value
Age (years)	41.7 ± 9.4	41.9 ± 9.6	0.842
Male (%)	58 (35.2%)	46 (30.7%)	0.200
BMI (kg/m^2^)	23.8 ± 3.2	23.6 ± 3.3	0.629
Max tumor size (cm)	0.80 ± 0.46	0.95 ± 0.67	0.093
Multiple lesions (%)	36 (21.8%)	45 (30.0%)	0.097
Hashimoto’s thyroiditis (%)	53 (32.1%)	49 (32.7%)	0.918
Total thyroidectomy (%)	68 (41.2%)	66 (44.0%)	0.617
Bilateral CCD (%)	54 (32.7%)	64 (42.7%)	0.069

CCD, central compartment dissection; INTP, intraoperative neural tunnel protecting; BMI, body mass index.

**Table 2:** Clinicopathologic characteristics in trans breast endoscopic thyroidectomy.

	INTP group (n = 94)	Control group (n = 95)	*p*-Value
Age (years)	33.2 ± 6.8	34.4 ± 7.3	0.245
Male (%)	3 (3.2%)	7 (7.4%)	0.200
BMI (kg/m^2^)	21.5 ± 2.6	22.0 ± 3.3	0.216
Max tumor size (cm)	0.83 ± 0.78	0.76 ± 0.51	0.459
Multiple lesions (%)	21 (22.3%)	19 (20.0%)	0.694
Hashimoto’s thyroiditis (%)	36 (38.3%)	28 (29.5%)	0.200
Total thyroidectomy (%)	25 (26.6%)	16 (16.8%)	0.104
Bilateral CCD (%)	22 (23.4%)	15 (15.8%)	0.187

CCD, central compartment dissection; INTP, intraoperative neural tunnel protecting; BMI, body mass index.

**Table 3:** Clinicopathologic characteristics in transoral endoscopic thyroidectomy.

	INTP group (n = 200)	Control group (n = 225)	*p*-Value
Age (years)	41.3 ± 9.1	41.4 ± 8.9	0.893
Male (%)	75 (37.5%)	83 (36.9%)	0.896
BMI (kg/m^2^)	24.2 ± 3.5	23.8 ± 3.7	0.255
Max tumor size (cm)	1.42 ± 0.39	1.48 ± 0.43	0.098
Multiple lesions (%)	60 (30.0%)	79 (35.1%)	0.262
Hashimoto’s thyroiditis (%)	66 (33.0%)	87 (38.7%)	0.224
Total thyroidectomy (%)	97 (48.5%)	116 (51.6%)	0.529
Bilateral CCD (%)	83 (41.5%)	112 (49.8%)	0.087

CCD, central compartment dissection; INTP, intraoperative neural tunnel protecting; BMI, body mass index.

### Effect of Recurrent Laryngeal Nerve Protection

EMG changes during the surgery and the complaints of hoarseness and laryngoscope examinations postoperatively were recorded (**Tables 4**–**6**). Compared to the control group, the rates of EMG changes were significantly decreased in the INTP group in trans breast endoscopic thyroidectomy (1.1% vs. 8.4%, *p* < 0.05). There were no significant differences in the rates of EMG changes of the INTP group in open (3.0% vs. 8.7%, *p* = 0.064) and transoral endoscopic (7.0% vs. 11.1%, *p* = 0.143) thyroidectomy. No permanent injury to the RLN injury was seen in the INTP group and the control group. The rate of transient hoarseness was significantly decreased in the INTP group compared with the control group in open (1.2% vs. 5.3%, *p* < 0.05) and transoral endoscopic (1.5% vs. 5.8%, *p* < 0.05) thyroidectomy. Rates of transient hoarseness of the INTP group were also decreased in trans breast endoscopic thyroidectomy (1.1% vs. 4.2%, *p* = 0.178), without significant difference.

**Table 4:** Effective assessment of INTP in open thyroidectomy.

	INTP group (n = 165)	Control group (n = 150)	*p*-Value
EMG changes (%)	5 (3.0%)	13 (8.7%)	0.064
Hoarseness (%)	2 (1.2%)	8 (5.3%)	0.037
Postoperative PTH (pg/ml)	35.7 ± 17.4	35.3 ± 17.1	0.837
Postoperative calcium	2.13 ± 0.12	2.15 ± 0.11	0.086
Numbness in limbs (%)	18 (10.9%)	16 (10.7%)	0.945
Total number of CLN	7.79 ± 4.63	7.19 ± 3.99	0.225
Number of metastatic CLN	1.05 ± 1.91	1.23 ± 1.75	0.406
WBC (×10^9^/L)	9.9 ± 8.5	8.6 ± 2.7	0.086
Postoperative CRP (mg/L)	8.0 ± 10.1	8.3 ± 7.9	0.826
Operative time (min)	67.6 ± 28.2	74.6 ± 31.8	0.064
Hospital stay (days)	3.2 ± 1.1	3.3 ± 1.1	0.392
Cosmetic satisfaction	6.19 ± 1.22	5.98 ± 0.97	0.160

CLN, central lymph nodes; INTP, intraoperative neural tunnel protecting; EMG, electromyography; PTH, parathyroid hormone; WBC, white blood cell; CRP, C-reactive protein.

**Table 5:** Effective assessment of INTP in trans breast endoscopic thyroidectomy.

	INTP group (n = 94)	Control group (n = 95)	*p*-Value
EMG changes (%)	1 (1.1%)	8 (8.4%)	0.018
Hoarseness (%)	1 (1.1%)	4 (4.2%)	0.178
Postoperative PTH (pg/ml)	38.7 ± 19.4	41.2 ± 16.2	0.329
Postoperative calcium	2.10 ± 0.10	2.13 ± 0.11	0.052
Numbness in limbs (%)	17 (18.1%)	10 (10.5%)	0.138
Total number of CLN	7.69 ± 5.71	7.35 ± 4.25	0.735
Number of metastatic CLN	0.83 ± 1.61	0.92 ± 1.52	0.706
WBC (×10^9^/L)	8.7 ± 2.5	8.8 ± 2.5	0.741
Postoperative CRP (mg/L)	9.6 ± 6.7	8.7 ± 5.9	0.369
Operative time (min)	124.0 ± 50.2	120.7 ± 44.4	0.635
Hospital stay (days)	3.2 ± 1.1	3.4 ± 1.1	0.298
Cosmetic satisfaction	7.79 ± 0.77	7.69 ± 0.70	0.390

CLN, central lymph nodes; INTP, intraoperative neural tunnel protecting; EMG, electromyography; PTH, parathyroid hormone; WBC, white blood cell; CRP, C-reactive protein.

**Table 6:** Effective assessment of INTP in transoral endoscopic thyroidectomy.

	INTP group (n = 200)	Control group (n = 225)	*p*-Value
EMG changes (%)	14 (7.0%)	25 (11.1%)	0.143
Hoarseness (%)	3 (1.5%)	13 (5.8%)	0.021
Postoperative PTH (pg/ml)	33.1 ± 17.1	30.0 ± 16.3	0.053
Postoperative calcium	2.14 ± 0.13	2.09 ± 0.13	< 0.01
Numbness in limbs (%)	27 (13.5%)	34 (15.1%)	0.636
Total number of CLN	9.42 ± 5.79	9.94 ± 6.32	0.377
Number of metastatic CLN	1.74 ± 2.41	2.13 ± 2.63	0.110
WBC (×10^9^/L)	8.9 ± 2.5	9.8 ± 5.3	0.047
Postoperative CRP (mg/L)	6.7 ± 5.9	8.7 ± 6.9	< 0.01
Operative time (min)	91.9 ± 33.3	92.4 ± 33.6	0.883
Hospital stay (days)	3.4 ± 1.2	3.4 ± 1.2	0.874
Cosmetic satisfaction	7.88 ± 0.74	7.76 ± 0.76	0.100

CLN, central lymph nodes; INTP, intraoperative neural tunnel protecting; EMG, electromyography; PTH, parathyroid hormone; WBC, white blood cell; CRP, C-reactive protein.

### Parathyroid Hormone and Calcium Assessment

The levels of PTH and serum calcium were recorded at day 1 following surgery. The PTH levels were similar between the INTP group and the control group in open (35.7 ± 17.4 vs. 35.3 ± 17.1, *p* = 0.837), trans breast (38.7 ± 19.4 vs. 41.2 ± 16.2, *p* = 0.329), and transoral (33.1 ± 17.1 vs. 30.0 ± 16.3, *p* = 0.053) thyroidectomy. Serum calcium levels in the INTP group (2.14 ± 0.13) were significantly higher than in the control group (2.09 ± 0.13) in transoral endoscopic thyroidectomy. On the other hand, there was no difference in the levels of calcium between the two groups in open (2.13 ± 0.12 vs. 2.15 ± 0.11, *p* = 0.086) and trans breast endoscopic (2.10 ± 0.10 vs. 2.13 ± 0.11, *p* = 0.052) thyroidectomy. The incidences of events of numbness in limbs were similar between the INTP group and the control group in open (10.9% vs. 10.7%, *p* = 0.945), trans breast (18.1% vs. 10.5%, *p* = 0.138), and transoral (13.5% vs. 15.1%, *p* = 0.636) endoscopic thyroidectomy.

### Influence of Central Compartment Dissection

We recorded the total number of central lymph nodes (CLNs) and metastatic CLNs (**Tables 4** **–6**). The total number of CLNs was similar between the INTP group and the control group in open (7.79 ± 4.63 vs. 7.19 ± 3.99, *p* = 0.225), trans breast (7.69 ± 5.71 vs. 7.35 ± 4.25, *p* = 0.735), and transoral (9.42 ± 5.79 vs. 9.94 ± 6.32, *p* = 0.377) endoscopic thyroidectomy. No differences were seen between the two groups in the number of metastatic CLNs in open (1.05 ± 1.91 vs. 1.23 ± 1.75, *p* = 0.406), trans breast (0.83 ± 1.61 vs. 0.92 ± 1.52, *p* = 0.706), and transoral (1.74 ± 2.41 vs. 2.13 ± 2.63, *p* = 0.110) endoscopic thyroidectomy.

### Influence of Postoperative Inflammatory Response

WBC and CRP were recorded to assess the postoperative inflammatory response. Compared with the control group, WBC levels in the INTP group were significantly decreased in transoral endoscopic thyroidectomy (8.9 ± 2.5 vs. 9.8 ± 5.3, *p* < 0.05). No significant differences were seen in WBC levels between the INTP group and the control group in the open (9.9 ± 8.5 vs. 8.6 ± 2.7, *p* = 0.086) and trans breast endoscopic (8.7 ± 2.5 vs. 8.8 ± 2.5, *p* = 0.741) thyroidectomy. Compared with the control group, CRP levels were significantly decreased in the INTP group in transoral endoscopic thyroidectomy (8.7 ± 6.9 vs. 6.7 ± 5.9, *p* < 0.05). CRP levels between the two groups were similar in open (8.0 ± 10.1 vs. 8.3 ± 7.9, *p* = 0.826) and trans breast endoscopic (9.6 ± 6.7 vs. 8.7 ± 5.9, *p* = 0.369) thyroidectomy.

### Operative Assessment and Follow-Up

Operative time, postoperative hospital stay, and cosmetic satisfaction were recorded to assess the surgical effects (**Tables 4**–**6**). The cosmetic satisfaction was assessed at 3 months post-surgery. The duration of surgery was similar between the INTP group and the control group in open (67.6 ± 28.2 vs. 74.6 ± 31.8, *p* = 0.064), trans breast (124.0 ± 50.2 vs. 120.7 ± 44.4, *p* = 0.635), and transoral (91.9 ± 33.3 vs. 92.4 ± 33.6, *p* = 0.883) endoscopic thyroidectomy. It was similar in the comparison of postoperative hospital stays between the two groups in open (3.2 ± 1.1 vs. 3.3 ± 1.1, *p* = 0.392), trans breast (3.2 ± 1.1 vs. 3.4 ± 1.1, *p* = 0.298), and transoral (3.4 ± 1.2 vs. 3.4 ± 1.2, *p* = 0.874) endoscopic thyroidectomy. No differences were seen in cosmetic satisfaction between the two groups in open (6.19 ± 1.22 vs. 5.98 ± 0.97, *p* = 0.160), trans breast (7.79 ± 0.77 vs. 7.69 ± 0.70, *p* = 0.390), and transoral (7.88 ± 0.74 vs. 7.76 ± 0.76, *p* = 0.100) endoscopic thyroidectomy.

## Discussion

EBDs have been widely adopted for surgical hemostasis given their effective blood-loss reduction and easy application (12). However, EBDs are also controversial since they might lead to potential nerve damage due to their lateral thermal spread during activation and high temperature on the head during activation (13). Furthermore, the “liquid–gas flow” generated by EBDs can also be a risk factor for RLN thermal injury (9, 14). RLN thermal injury is severe and can lead to paralysis of the VC (15, 16). Notably, it would be quite effective to isolate the RLN with lateral thermal spread and liquid–gas flow. Hence, we introduced a new strategy of INTP and evaluated its effects of protecting the RLN in open, trans breast, and transoral endoscopic thyroidectomy.

Endoscopic gauze was found to be useful for protecting the nerve from potential thermal risk factors of lateral heat conduction and thermal liquid–gas flow. This helped minimize the incidence of RLN thermal damage during thyroidectomy. Moreover, a standardized strategy of INTP utilizing gauze was introduced in open, trans breast, and transoral endoscopic thyroidectomy. During surgery, the segment of the RLN was exposed at the inferior pole of the thyroid lobe in open and trans breast endoscopic thyroidectomy. On the other hand, it had been exposed at the superior pole in transoral endoscopic thyroidectomy. The endoscopic gauze was stuffed into the neural tunnel. This helped to protect the RLN from lateral heat conduction and thermal liquid–gas flow. The gauze was placed in the gap of the RLN and EBDs consistently until the thyroid lobe was resected completely.

A systematic strategy of INTP was firstly introduced in open, trans breast, and transoral endoscopic thyroidectomy. Our center is experienced in endoscopic thyroidectomy, including trans breast, transoral, and trans axillary approaches, and robotic-assisted endoscopic thyroid surgeries (17, 18). Between January 2019 and June 2020, hundreds of patients in whom open, trans breast, and transoral endoscopic thyroidectomy were performed were enrolled to analyze if INTP helped in protecting the RLN. In this current retrospective analysis, the clinicopathologic characteristics between the INTP group and the control group were similar. This included age, gender index, BMI, tumor size, multiple lesions rates, Hashimoto’s thyroiditis rates, total thyroidectomy, and bilateral CCD rates in open, trans breast, and transoral endoscopic thyroidectomy.

The strategy of INTP was found to be effective in RLN protection during thyroidectomy. There was a significant decrease in the rate of postoperative hoarseness in the INTP group, especially in the open and transoral endoscopic thyroidectomy. There was also a significant decrease in EMG changes in the INTP group in trans breast endoscopic thyroidectomy. Interestingly, consistent with the previous study (19), while EMG changes were seen in some patients during surgery, the symptom of hoarseness was not seen in them postoperatively. It seems that the implementation of gauze reduced the degree of nerve tractive or thermal injury, which manifested as EMG changes, but the conduction ability can be recovered in a short period postoperatively. Endoscopic gauze covered on the surface of the RLN effectively blocked the lateral thermal spread and thermal flows generated by EBDs.

INTP is also beneficial in reducing the postoperative inflammatory response in transoral endoscopic thyroidectomy. In a transoral endoscopic thyroidectomy, there was a significant decrease in the postoperative WBC count and CRP levels in patients in the INTP group. Small workspace during the whole surgery is the special characteristic of transoral endoscopic thyroidectomy (20). In this situation, the distance between the important tissues and EBDs is considered to be too small, which increased the incidences of surgical complications. In the INTP group, gauze was implemented to supply a barrier that is useful for protecting important tissues from thermal injuries or other risk factors generated by EBDs. Thus, using INTP helped reduce the inflammatory response. Furthermore, the postoperative PTH serum calcium was significantly higher in the INTP group compared to the control group. This indicated that using the INTP strategy may help protect the parathyroid glands in transoral endoscopic thyroidectomy.

IONM is useful in identifying the RLN during thyroidectomy (21). Our center firstly implemented IONM in trans breast and transoral endoscopy thyroidectomy (17, 22). However, due to the lack of a standardized surgical procedure, there was no decrease in the incidence of RLN injury in the open and endoscopic thyroid surgeries (23). We introduced a new strategy of INTP in the current study, which was found to be useful in protecting the RLN in open, trans breast, and transoral endoscopic thyroidectomy. Since the materials and techniques of INTP can be easily acquired, we believe it can be widely spread in multiple medical centers. Moreover, the INTP strategy may also be helpful in protecting important tissues by creating a separation barrier in transoral endoscopic thyroidectomy whose effects include reducing the postoperative inflammatory response and protecting the parathyroid glands.

The INTP strategy would not increase adverse effects, such as extending operative time. Moreover, there was no difference in the total number of CLN and metastatic CLN between the two groups. The results of hospital stay and cosmetic satisfaction were also similar between the INTP group and the control group. These results suggested that the use of the INTP strategy did not influence surgical outcomes. However, our study had some limitations. Firstly, it was a retrospective study, where we only enrolled those patients who had a pathological diagnosis of PTC from January 2019 to June 2020 at our center. Secondly, the surgery choices of ipsilateral lobectomy or total thyroidectomy, and central node dissections were according to Chinese guidelines. Additionally, all surgeries were performed within a period of 13–31 months, and the follow-up time was not long enough to observe tumor recurrence.

## Conclusion

The new strategy of INTP was introduced in this study, which can be easily acquired in open, trans breast, and transoral endoscopic thyroidectomy. Incorporating the strategy of INTP may effectively protect the RLN from thermal liquid–gas flow and other risk factors and also be helpful in decreasing the incidence of postoperative nerve injury. Additionally, it was also seen that INTP helped protect important tissues by creating a separation barrier in transoral endoscopic thyroidectomy, thereby decreasing the postoperative inflammatory responses and protecting the parathyroid glands.

## Data Availability Statement

The original contributions presented in the study are included in the article/supplementary material. Further inquiries can be directed to the corresponding authors.

## Ethics Statement

Written informed consent was obtained from the individual(s) for the publication of any potentially identifiable images or data included in this article.

## Author Contributions

All authors participated in the design, execution, and interpretation of the study. PW, YW, and XY conceived and designed the study. XY, YL, CL, YJ, and ZL performed the majority of studies and analyzed data. XY and QH contributed to the analyses and interpretation of the study. XY and CL wrote the manuscript. PW and YW approved the final, submitted manuscript.

## Funding

This study is supported by the Health Innovation Talents Project of Zhejiang Province (2021RC004) and Basic Public Welfare Research Project of Zhejiang Province (LGF22H070002).

## Conflict of Interest

The authors declare that the research was conducted in the absence of any commercial or financial relationships that could be construed as a potential conflict of interest.

## Publisher’s Note

All claims expressed in this article are solely those of the authors and do not necessarily represent those of their affiliated organizations, or those of the publisher, the editors and the reviewers. Any product that may be evaluated in this article, or claim that may be made by its manufacturer, is not guaranteed or endorsed by the publisher.
